# Basic Helix-Loop-Helix (bHLH) Transcription Factors Regulate a Wide Range of Functions in Arabidopsis

**DOI:** 10.3390/ijms22137152

**Published:** 2021-07-01

**Authors:** Yaqi Hao, Xiumei Zong, Pan Ren, Yuqi Qian, Aigen Fu

**Affiliations:** Chinese Education Ministry’s Key Laboratory of Western Resources and Modern Biotechnology, Key Laboratory of Biotechnology Shaanxi Province, College of Life Sciences, Northwest University, China P.R. 229 North Taibai Road, Xi’an, Shaanxi 710069, China; yaqihao@nwu.edu.cn (Y.H.); zongxiumei@stumail.nwu.edu.cn (X.Z.); renpan5521@163.com (P.R.); qyq916@163.com (Y.Q.)

**Keywords:** basic helix-loop-helix (bHLH), transcription factor, cross-talk, plant growth and development

## Abstract

The basic helix-loop-helix (bHLH) transcription factor family is one of the largest transcription factor gene families in Arabidopsis thaliana, and contains a bHLH motif that is highly conserved throughout eukaryotic organisms. Members of this family have two conserved motifs, a basic DNA binding region and a helix-loop-helix (HLH) region. These proteins containing bHLH domain usually act as homo- or heterodimers to regulate the expression of their target genes, which are involved in many physiological processes and have a broad range of functions in biosynthesis, metabolism and transduction of plant hormones. Although there are a number of articles on different aspects to provide detailed information on this family in plants, an overall summary is not available. In this review, we summarize various aspects of related studies that provide an overview of insights into the pleiotropic regulatory roles of these transcription factors in plant growth and development, stress response, biochemical functions and the web of signaling networks. We then provide an overview of the functional profile of the bHLH family and the regulatory mechanisms of other proteins.

## 1. Introduction

Plants frequently encounter a wide array of environmental stresses, including drought, cold, salinity, UV radiation, ozone and other abiotic stresses through their lifecycle, which severely affect plant growth, development and crop yield. To cope with those problems, plants adapt to the different harsh conditions through transcription factors that regulate the expression of a series of target genes. The basic helix-loop-helix (bHLH) protein family is one of the largest transcription factor gene families in Arabidopsis thaliana, and contains a highly conserved amino acid motif that can be found throughout eukaryotic organisms. They were originally identified in animals, and found to play important roles in diverse regulatory networks, via the functions of DNA binding and dimerization [1,2]. The binding and dimerization networks of those proteins also have diverse functions in plants, including in multiple aspects of plant growth and development, which were characterized in recent years.

The bHLH superfamily, named after its highly conserved alkaline/helix-loop-helix domain, is the second largest transcription factor family and is widely present in animals and plants [3,4]. The bHLH domain is composed of approximately 60 conserved amino acid residues and consists of two conserved motifs, namely, a basic region and helix-loop-helix region (HLH region). The basic region participates in DNA binding to E-box (usually CANNTG) or G-box (CACGTG) motif in their target genes, and the HLH region, which is composed of two alpha helices containing hydrophobic residues, is required for dimerization to change the expression of target genes involved in various signaling pathways. Based on their phylogenetic relationship and DNA binding function, the bHLH members in eukaryotes have been classified into six main groups (named A, B, C, D, E and F) [5]. In brief, Group A can specifically bind to the E-box core sequence, Group B prefers to bind the G-box, and Group C binds to the ACTTG or GCGTG sequences [6,7]. Group D lacks a typical basic region and mainly participates in heterodimerization with other bHLH family proteins [8]. Group E can preferentially bind to the CACGNG sequence, and the Group F members can bind to specific DNA target sequences [9,10,11]. At least 162 members of bHLH proteins have been identified in Arabidopsis [12,13,14], which could be clustered into 12 distinctive subfamilies, namely bHLH I to XII [13,14]. Though bHLH proteins in Arabidopsis are quite divergent, the most abundant type of Arabidopsis bHLHs are more closely related to the phylogenetic group B of eukaryotic bHLHs, which is proposed to be the ancestral bHLH type [14,15]. Arabidopsis also contains some atypical bHLH proteins, which lack the basic region and are referred as AtHLHs. However, they do not share a close sequence similarity to the group D proteins in animals [4,14].

As a superfamily, bHLHs have been characterized in Arabidopsis over the years, and members of this family members play vital roles in plant growth, development, light signal transduction and stress responses [16,17,18,19,20]. They are also involved in the crosstalk of hormones signaling, including abscisic acid (ABA), jasmonic acid (JA), brassinosteroid (BR), salicylic acid (SA) and ethylene (ET) [1,4,13,21], and they are pivotal for plant growth and survival in the environment.

**Figure 1:**
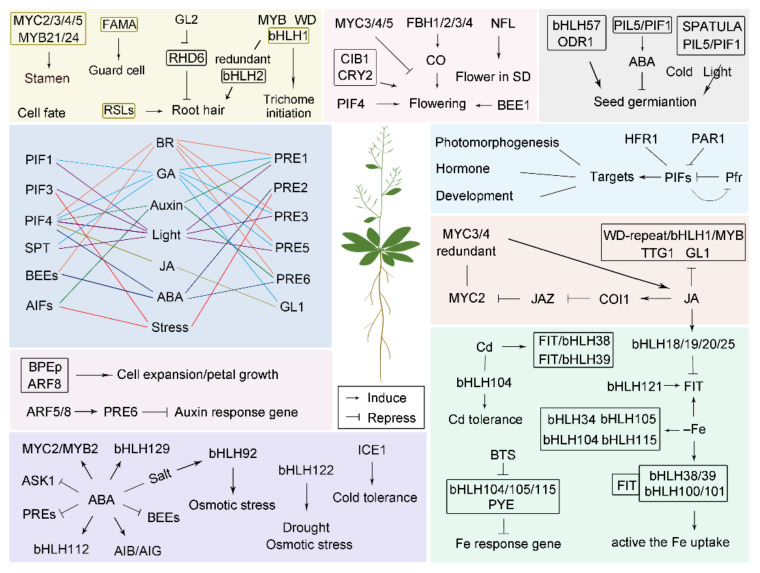
bHLHs functions in various signaling pathways.

To date, many excellent reviews have covered the roles of bHLH transcription factors in plants in different contexts, but an overall summary is not available. Thus, we focused mainly on summarizing the knowledge on bHLH functions in various essential processes, such as how bHLH proteins mediate plant growth and development, including flowering time, seed germination and cell fate determination; how they are involved in maintaining iron homeostasis in plants, how they respond to light, stress, phytohormones and the crosstalk among these factors; and how they perform the functions in other processes, such as senescence and anthocyanin biosynthesis (Figure 1 and Table 1).

## 2. Functions of bHLH Factors in Plant Growth and Development in Arabidopsis

### 2.1. Roles in Seed Germination

Seed dormancy and germination are critical processes in the lifespan of plants, which are mediated by various external environmental factors such as temperature, light and humidity, and internal factors, such as phytohormones. Two type of phytohormones, ABA and gibberellin (GA), are crucial regulators of seed dormancy and germination. ABA enhances seed dormancy, whereas GA breaks dormancy and promotes germination. The transcription factor bHLH57 is involved in ABA-regulated the seed dormancy process. NCED (9-cis-epoxycarotenoid dioxygenase) is considered to catalyze the first dedicated step in ABA biosynthesis [22], ODR1 (reversal of rdo5, an ortholog of the rice seed dormancy 4 (Sdr4)) protein interact with bHLH57 in a complex to inhibit NCED gene transcription [23,24].

In addition to phytohormones, photoreceptors also regulate seed dormancy and germination. Phytochrome interacting factors (PIFs), a group of typical of bHLH proteins, could interact with diverse groups of factors to integrate external environmental and internal signals, and further control seed germination, shade avoidance and crosstalk of plant hormones and the clock-derived signaling pathway [25,26]. The phytochrome-interacting bHLH protein PIL5 (PIF3-like 5 PIF1/bHLH15) is a repressor of seed germination, acting by reducing GA level in the dark; phytochromes accelerate the PIL5 degradation and increase the level of bioactive GA in seeds upon exposure to light. In addition, PIL5 activates the expression of the ABA synthesis gene and maintains a high level of ABA in the dark to inhibit seed germination [27]. Another light stable bHLH transcription factor SPATULA (SPT) also mediates seed germination, by interacting with PIL5 in response to light and temperature [28,29].

### 2.2. Functions in the Flowering Time Control

It is critical for plants to manage their flowering time, which is regulated by an intricate network of molecular signaling and controlled by various environmental factors such as photoperiod and temperature. The CONSTANS (CO) protein functions as an essential component for transforming biological clock signals into flowering signals to initiate plant flowering. Four bHLH related proteins (flowing bHLHs), namely, FBH1, FBH2, FBH3 and FBH4, are transcriptional activators of the *CO* gene; they bind to the E-box cis-elements in the promoter of *CO* and then positively regulate the CO-mediated flowering time [30]. Cryptochromes (CRY1/2) are blue light receptors that inhibit hypocotyl elongation and control floral initiation [31]. CRYPTOCHROME-INTERACTING BASIC-HELIX-LOOP-HELIX 1 (CIB1) is a CRY2-interacting bHLH protein that promotes the FLOWERING LOCUS T (FT) transcription; CRY2, CIB1 and CO can form a protein complex in response to blue light and then promote floral initiation [32,33].

Recently, a bHLH transcription factor, named as NO FLOWERING IN SHORT DAY (NFL) was shown to be necessary for the promotion of flowering specifically under short-day (SD) conditions, and *nfl* mutants did not flower under SD conditions but were similar to wild type under long-day (LD) conditions [34]. Additionally, three myelocytomatosis (MYC) proteins redundantly regulate flowering under both LD and SD conditions. Although MYC4 (bHLH4) is more important in mediating flowering than MYC3 (bHLH5), MYC2/3/4 are required in the JA pathway for regulating the flowering time by inhibiting FT transcription in Arabidopsis [35]. Moreover, the Brassinosteroid Enhanced Expression1 (BEE1, bHLH44) protein is stabilized under blue light; this protein is an integrator regulating photoperiodic flowering [36]. 

In Arabidopsis, temperature also affects the flowering time, and high temperature (29 °C) not only induces rapid hypocotyl elongation but also results in early flowering. In addition, the central integrator bHLH transcription factor phytochrome-interacting factor 4 (PIF4) can accelerate the flowering process by activating FT at high temperature conditions [37]. In addition to regulating the flowering time, bHLH family members are involved in the flower organ development. SPATULA (SPT) encodes a bHLH transcription factor and plays a role in floral morphogenesis processes as previously described [38]. 

In summary, the flowering time is strictly controlled by an intricate network, and bHLH family members act together with many other proteins to allow plants to flower at suitable time and under favorable environments.

### 2.3. Functions on Cell Fate Determination

Plants process a cell determination mechanism for formatting specific cell types, and this system relies on the expression of different genes in a proper spatiotemporal manner. Many transcription factors participate in this critical process, including a number of bHLH transcription factors, which play essential roles in the root and shoot cell fate determination [39]. During the root development, two types of cells arise from the epidermis: root hair cells and non-hair cells. Root hair cells produce an outside long tubule from a specialized root surface area and have functions in absorbing nutrition and water, and also in interacting with microbes.

The bHLH VIIIc subfamily member, bHLH83 (root hair defective 6, RHD6), is a transcription factor that has fewer root hairs and functions in the inhibition root hair formation in cortical cells [13,39], and another bHLH protein, ROOT HAIR DEFECTIVE 6-Like 1 (RSL1), functions redundantly with RHD6 to modulate the root hair development [40]. RSL2, a homolog of RSL1, is also required for the normal root hair formation, which is controlled by RHD6 and RSL1 [41]. Additional genes, such as *RSL3/4* and *LRL3* (Lj-RHL1-LIKE3) also act as downstream of RHD6 and RSL1 to promote the root hair and cell differentiation [41,42,43]. Moreover, RSL4 is suppressed by GLABRA2 (GL2) through ZINC FINGER PROTEIN1 (ZP1) [44]. GL2, a member of homeodomain-leucine zipper (HD-ZIP) protein, affects the epidermal cell fate including in trichomes, root hair and the seed coat. It represses the transcription of RHD6 to inhibit hair formation in N-cells (non-root hair cells), leading to the expression of non-hair genes [42,45]. The bHLH transcription factor GL3 (GLABRA3, bHLH1) has similar functions, regulating its own transcription and downstream target genes to trigger the trichome initiation pathways, ENHANCER OF GLABRA3 (EGL3, bHLH2) plays partially redundant roles with GL3 in root hair [46,118].

Furthermore, ET is involved in root hair initiation and elongation, and ETHYLENE INSENSITIVE 3 (EIN3) and its homolog EIN3-like1 (EIL1) can coordinate with RHD6/RSL1 to upregulate RSL4 and then participate in root epidermis development [119]. Another bHLH complex named MYB-bHLH-WD40 was proposed to regulate the guard cell and root hair differentiation [47].

In addition to their roles in the root epidermis formation, the bHLH family is also involved in other cell fate related processes, including stamen and stomatal development. The IIIe bHLH transcription factor MYC5 (bHLH28) has redundant functions with MYC2 (bHLH6), MYC3 (bHLH5) and MYC4 (bHLH4), which interact with MYB21 and MYB24 to form the bHLH-MYB complex to regulate the stamen development [48]. In addition, FAMA (bHLH97) is specifically expressed in the stomata and has functions in halting proliferative division and promoting guard cells [120].

Overall, the establishment of this specification mechanism is complicated and also influenced by several plant growth regulators, including hormones such auxin (indole-3-acetic acid, IAA), ET, JA [55,119].

## 3. Functions in Environmental Response

### 3.1. Functions in Plant Mineral Nutrition and Abiotic Stress

Iron is an indispensable mineral micronutrient for photosynthesis and respiration in plants. Low concentration of effective iron in soil leads to the iron deficiency in plants, conversely, too much iron can have a toxic effect. To cope with this problem, plants have developed a series of sophisticated regulatory systems to control the iron uptake and maintain the Fe homeostasis. Among the regulatory mechanisms, bHLH transcription factors play important roles in this complicated process [49,92].

Four closely related clade Ib bHLH genes, namely, bHLH38, bHLH39 [50], bHLH100 and bHLH101 [121], have been characterized as key transcription factors regulating the Fe-deficiency response in Arabidopsis, and their transcript levels were shown to be up-regulated under Fe deficiency [50,121]. A single loss-of-function mutant of these genes did not show an obvious change in phenotype compared with wild-type because of functional redundancy in this subfamily. A triple loss-off function mutant of bHLH39, bHLH100 and bHLH101 showed severe leaf chlorosis only under Fe-deficient conditions [51]. bHLH29 (FER-like iron deficiency-induced transcription factor, FIT), an ortholog of FER (from the T3238FER line), acts as a central regulator to iron deficiency inducible genes in iron uptake and homeostasis [52,53,54]. Research has indicated that FIT interacts with bHLH38, bHLH39 [50], bHLH100 and bHLH101 [121] to form heterodimers that activate the transcription of genes involved in the Fe uptake system. Recent research has shown that bHLH121 can form homo or heterodimers and directly bind the *FIT* promoter to regulate its expression [122]. In addition, cadmium (Cd) stress is a severe heavy metal stress that limits plant growth, and FIT/bHLH38 or FIT/bHLH39 heterodimers can respond to Cd tolerance by affecting the transcriptional expression of downstream genes [56].

Four IVc bHLH transcription factors (bHLH34, bHLH104, bHLH105/ILR3 - IAA-LEUCINE RESISTANT3, and bHLH115) form homo- and heterodimers to regulate the Fe deficiency response and the Fe homeostasis [57,58,59,92]. Knockout of bHLH104 reduced Arabidopsis tolerance to Fe deficiency and suppressed the activation of Fe deficiency-inducible genes, whereas overexpression had the opposite effect. The bHLH034, homolog of bHLH104, also positively regulated the Fe deficiency response. The bHLH105, also named IAA-LEUCINE RESISTANT3 (ILR3), interacted with bHLH104, which plays an important role in maintaining the Fe homeostasis. Notably, a triple mutant deficient of bHLH34/104/105 is sensitive to Fe deficiency [59].

In the case of FIT, its function is required to regulate Fe uptake responses; bHLH47 (POPEYE, PYE) is another bHLH transcription factor that plays a major role in the Fe homeostasis. PYE interacts with bHLH104, bHLH105 and bHLH115 to bind to the promoters of several Fe response genes and repress their activity [60,61,62,123]. Interestingly, the bHLH104, bHLH105 and bHLH115 protein levels are posttranslationally controlled by BRUTUS (BTS), a hemerythrin E3 ligase, via the proteosomal degradation to negatively regulate the bHLH protein stability [61,124]. It was reported that the BTS paralogs, BTS LIKE1 (BTSL1) and BTSLIKE2 (BTSL2) act redundantly as negative regulators of the Fe starvation response, and the transcript levels are also controlled by FIT [125].

In most plants, Fe(II) is taken up by IRON REGULATED TRANSPORTER 1 (IRT1) in the root plasma membrane from soil under iron deficiency [63,126,127]. Moreover, ferric reduction oxidase 2 (FRO2) transfers electrons across the plasma membrane for iron reduction at the root surface. These are key genes regulating ferric reduction and iron transport in plants, and their transcription levels are upregulated by FIT and bHLH Ib heterodimers [50,121]. Furthermore, bHLH11 acts as a negative regulator to modulate Fe levels in Arabidopsis, and bHLH11 is highly expressed in roots and its expression is upregulated after plants are transferred to Fe sufficient conditions [64]. 

In addition to regulating the iron response, it was reported that bHLH104 loss-of-function mutants were sensitive to Cd stress, and the Cd tolerance was enhanced upon overexpression of bHLH104 [20]. In other words, bHLH104 positively regulates both Cd tolerance and Fe deficiency tolerance in Arabidopsis.

### 3.2. Functions in Stress Responses

Inducer of CBF Expression (ICE1, bHLH116) encodes an MYC-type bHLH transcription factors that directly binds the promoter of the C-Repeat Binding Factor/Dehydration-Responsive-Element-Binding protein (CBF/DREB1) protein-encoding gene and activates its transcription to improve cold tolerance through an ABA independent pathway [64]. The regulation is controlled by the binding of various downstream target cold and dehydration response genes that contain specific binding sites [65,128,129,130].

Other transcription factors, such as AIF2 (also named RITF1) and its partner RSA1 (SHORT ROOT IN SALT MEDIUM 1) regulate several gene for detoxification of reactive oxygen species (ROS), which are triggered by the salt stress [66].

Some proteins, such as bHLH122, can directly bind to the CYP707A3 promoter to repress its expression and enhance the ABA content, but these proteins were not induced by ABA treatment, indicating that they may function in ABA-independent or ABA-dependent ways [22,67].

As direct target genes of PIF4, JUNGBRUNNEEN1 (JUB1) and ORESARA1 (ORE1) have vital roles in salt stress response. PIF4 directly regulates ORE1 and JUB1, and is involved in regulating salt stress response [68,69,70].

## 4. Functions in the Response to Light and Phytohormones

Under specific conditions, multiple phytohormones and environmental factors are in constant crosstalk with each other to affect the plant growth and development. Based on previous studies, GA, IAA, BR, ET and light are usually considered to promote cell expansion in plant growth, while ABA, JA and SA are normally involved in the response to biotic and abiotic stresses. bHLH proteins that respond to plant hormones and environmental factors participate in various processes. They are able to interact with each other to cooperatively or antagonistically modulate the plant growth.

### 4.1. Response to Light Signaling

Light is an important factor that influences the plant growth and development, and many bHLH transcription factors are reported to participate in this process by inducing the expression of related downstream genes and various light-mediated effects.

PIFs interact with the active form (Pfr) of phytochrome to modulate growth, including in response to environmental signals such as light and stress and via other signaling pathways [73]. The PIF family belongs to the bHLH superfamily VII of transcription factors, which play central roles in the regulation of light signaling. To date, 15 PIF members have already been identified, and 7 PIF members have been shown to bind the Pfr form of phyB in Arabidopsis, while other members do not interact with the light-activated phytochrome [13,14,131,132,133]. Each PIF has individualized or redundant biological functions with other PIF proteins during various responses.

In the dark, four PIFs (PIF1/PIL5, PIF3, PIF4 and PIF5/PIL6) directly interact with Pfr to promote skotomorphogenesis by repressing photomorphogenesis. Loss-of-function of *pif* mutant showed phenotypes of reduced hypocotyl elongation. PIFs also interact with diverse groups of transcription factors to integrate external environmental and internal signals, including in seed germination, shade avoidance and crosstalk among plant hormones and clock-related signaling pathways [24,25]. A number of genes have been confirmed to be direct targets of PIF, thus mediating downstream light signaling networks through the PIFs.

PIF3 (bHLH8), the first identified member in the PIF family, acts as a regulator in the seedling de-etiolation and modulates both positive and negative response to phytochrome-mediated signaling [24,71]. PIF3 can also heterodimerize with the atypical basic bHLH protein HFR1 (long hypocotyl in far-red, bHLH26) to modulate phyA signaling [75]. In addition, PIF3 is involved in plant freezing response as a negatively regulator which can interact with EBF1 (EIN1-BINDING F-BOX1) through the CBF (C-REPEAT BINDING FACTOR) pathway [72].

PIF4 is a key transcription factor in the light signaling pathway; it interacts selectively with Pfr and negatively regulates phyB signaling in Arabidopsis [74], Phytochrome Rapidly Regulated 1 (PAR1, bHLH165) and its homolog PAR2 (bHLH166) lack the ability to directly interact with phytochromes that are rapidly induced by shade [76,77], while PAR1 directly interacts with PIF4 to form a heterodimer to inhibit PIF4 function in cell elongation [78]. Moreover, growing evidence indicates that PIF4 acts as central regulator, coordinating plant response to multiple environmental signals [134]. 

The PIL (PIF3-like proteins, PILs) family has six members, designated as PIL1, PIL2, PIL3, PIL4, PIL5 and PIL6. PIF1/PIL5 (bHLH15) is a component that negatively regulate the chlorophyll biosynthetic pathway, seeds germination and inhibit hypocotyl elongation in the dark. The activity of PIF1 was repressed by phyA and phyB in light, and regulated ABA signaling [79,80,81,82]. In addition, other PIFs, such as PIF5 (PIL6), PIF6 (PIL2) and PIF7, have been shown to interact with the Pfr form of phyB or phyA, which are involved in phytochrome signaling [83,84,85].

Taken together, the bHLH proteins PILs/PIFs are proposed to form heterodimers to regulate bHLH network activity and are central components that integrate multiple signals in response to light.

### 4.2. Functions in JA Signaling Pathway

The phytohormone jasmonate acid (JA) plays a vital role in the plant development and the response to various stresses. The presence of JA triggers the key protein jasmonate ZIM-domain (JAZ) to interact with Coronatine Insensitive 1 (COI1), part of the SCF^COI1^ ubiquitin E3 ligase complex. Then, JAZ proteins were degraded by the 26S protease, resulting in multiple transcription factors free from JAZ-mediated repression and further activating downstream JA-mediated responsive genes. The bHLH transcription factor MYC2 forms a signaling module COI1/JAZs/MYC2 to participate in the JA-induced signaling pathway. The homologs of MYC2, MYC3 (bHLH5) and MYC4 (bHLH4) are known to form homodimers/heterodimers, and they can also bind to the G-box involved in the JA signaling pathway but exhibit gene redundancy with MYC2 [86,87].

The bHLH transcription factor GLABRA3 (GL3, bHLH1) can form a WD-repeat/bHLH/MYB complex with TRANSPARENT TESTA GLABRA1 (TTG1) and the R2R3-MYB transcription factor GLABRA1 (GL1), which is repressed by JAZs and DELLAs, is responsible for trichome initiation [88,89,90,91].

Four subgroup IVa bHLH transcription factors (bHLH18, bHLH19, bHLH20 and bHLH25) can be induced by JA and inhibit the transcription of the FIT and Ib bHLH genes, which have been suggested to function redundantly in JA-mediated FIT protein degradation in the presence of JA or under iron deficient conditions via the 26S proteasome pathway [92].

The jasmonate-activated transcription factor MYC2 has also been found to interact with the key component in the ET signaling pathway EIN3 and its close homolog EIL1 to repressed its DNA binding activity and affect hook formation [135], suggesting that jasmonate and ET have antagonistic functions during apical hook development.

### 4.3. Functions in IAA Signaling Pathway

The plant hormone IAA has multiple roles in the plant growth and development, such as in cell division, cell elongation and cell differentiation, which are affected by the regulation of IAA response genes [93]. BIGPETALp (BPEp, bHLH31) is a bHLH transcription factor that can interact with AUXIN RESPONSE FACTOR8 (ARF8) to influence cell expansion and petal growth [94], PRE6 is a target of ARF5 and ARF8 that negatively regulates auxin response genes in Arabidopsis [95].

### 4.4. Roles in ABA Signaling Pathway

Plant are constantly under extrinsic abiotic/biotic environmental stresses, including cold, drought, high salinity, pathogen and extreme temperature. In response to these diverse stresses, plants have evolved sophisticated adaptation mechanisms. To date, several bHLH transcription factors have been reported to mediate abiotic and biotic stress signaling pathways to regulate plant responses in Arabidopsis in different ways. 

The plant hormone ABA plays a central role in a variety of physiological processes and environmental response involved in plant growth, including responses to drought, cold, heat and salinity stresses [136,137,138]. Several bHLH transcription factors, such as bHLH112, MYC2, AIB, AtAIG1, bHLH129 and bHLH92, have been reported to be involved in the regulation of ABA signaling via these processes [96,98,99,100].

A loss-of-function mutant of bHLH112, a transcriptional activator, displayed a late-flowering phenotype under long day conditions in Arabidopsis, and its transcript level was correlated with salt and drought tolerance. bHLH112 regulates gene expression by binding to E-box and GCG-box motifs in the gene promoters and then mediate multiple physiological response to improve stress tolerance [96,97].

ABA-inducible bHLH-type transcription factor (AIB) and ABA-inducible gene (AIG1) are ABA-induced genes, the proteins contain a bHLH type DNA binding domain and play a positive role in ABA signaling in Arabidopsis [98,99]. MYC2 and MYB2 have been shown to be transcription activators that function in the ABA signal transduction pathway by directly regulating the expression of ABA response genes [17]. In addition, MYC2 can also be involved in JA [139,140,141].

The expression level of bHLH129 is reduced upon exogenously application of ABA, and bHLH129 regulates the expression of several ABA signaling component genes [100] to promote root elongation. 

PREs are involved in plant growth and development and are also involved in the regulation of ABA mediated salt responses in Arabidopsis. Some PREs are ABA responsive genes; their expression levels are decreased under ABA treatment, and this response has functions in regulating plant growth and environmental stimuli [101].

ABA induces stomatal closure and changes the expression of numerous genes to adapt to drought stress, ABA-responsive kinase substrate (AKS1), also a bHLH transcription activator, is inhibited by ABA through phosphorylated to form monomer by SNF1-related protein kinase 2 (SnRK2) in Arabidopsis guard cells [142,143].

In addition, the BEE transcription factor family members (BEE1/2/3) regulate plant responses to abiotic stress. BEE genes are strongly repressed by ABA and are redundant negative regulators of physiological responses to abiotic stress, whereas the BEE2 dimerized protein IBH1 is a positive modulator that improves salt and drought tolerance [102]. NaCl-induced expression of bHLH92 confers tolerance to salt and osmotic stress which is partially dependent on ABA and SALT OVERLY SENSITIVE 2 (SOS2) [103].

### 4.5. The Cross-Talk between Light and Phytohormones

Some bHLH transcription factors are involved in signal transduction networks mediated by plant hormones. For example, several genes are involved in light and GA signaling pathways, such as PIL5 and SPT, which also belong to the bHLH transcription factor family [27,80]. Moreover, many bHLH transcription factors have been reported to be involved in the regulation of BRs, ABA and IAA signaling pathways. Paclobutrazol Resistance (PREs) proteins are atypical bHLH transcription factors that lack the DNA binding domain but can dimerize with bHLH factors to inhibit DNA binding [107,144]. PREs participate in various hormone-, temperature- and light-responsive signaling pathways to regulate plant growth and development in many ways [104,105,107,145]. Briefly, PRE1 (bHLH136), PRE5 (bHLH164) and PRE6 (bHLH163) are direct targets of PIF4 and are involved in the regulation of the light, GA and BR signaling pathways [25,104]. PRE1 regulates cell elongation in Arabidopsis together with ARF6 [105]. PRE3 (bHLH135), also named Activation-Tagged BRI1 Suppressors 1 (ATBS1), can suppress the BR insensitive 1 (bri1) phenotype, and its mutant shows an auxin-related phenotype [16]. PRE4 (bHLH161, BNQ3) mutant shows a light-related phenotype, including pale-green sepals, decreased chlorophyll levels and late flowering [106]. PRE2 (bHLH134) and PRE6 (bHLH163) are ABA response genes that affect plant sensitivity to ABA, indicating that some PREs are involved in ABA and salts responses [95].

Several atypical bHLH proteins, such as Arabidopsis ILI1 binding bHLH1 (IBH1) and ATBS1 Interacting Factors (AIFs), negatively regulate cell elongation in Arabidopsis [107,108]. Activators for cell elongation (ACEs) can promote cell elongation, while IBH1 interacts with ACEs to inhibit their functions in the induction of cell elongation. Another bHLH protein, homolog of BEE2 interacting with IBH1 (HBI1), is involved in BR-mediated growth to promote cell elongation [104]. Functional analysis showed that bHLH proteins have related functions but with different mechanisms to regulate cell elongation. In these processes, bHLHs proteins are mostly dependent on multiple phytohormones including BR, GA, IAA and light signaling [108,146].

BEE1 (bHLH44), BEE2 (bHLH58) and BEE3 (bHLH50) are functionally redundant bHLH transcription factors for which expression is induced by BL treatment, indicating that they function in the early response to BRs and their expression is required by ABA [18]. In brief, the BEE1 protein is stabilized under blue light, which is an integrator to regulate photoperiodic flowering [109]. BEE2 and CIB1 negatively regulate immunity and are functionally redundant with HBI1 in plant immunity [104,110]. Moreover, BEEs positive regulate shade avoidance syndrome with BES1-INTERACTING MYC-LIKE protein (BIMs) [111,112].

## 5. Functions in Other Aspects of Plant Biology

In Arabidopsis, bHLH members are also involved in other aspects of plant growth and development, such as senescence and the anthocyanin biosynthesis pathway. These aspects are only briefly discussed here.

In plants, senescence is triggered by developmental and environmental factors. The IIId bHLH transcription repressors (bHLH3, bHLH13, bHLH14 and bHLH17) can bind to the promoter of senescence-associated gene 29 (SAG29) to repress MYC2-activated leaf senescence [47]. PIFs can promote leaf senescence during age-triggered and dark-induced processes [113].

bHLH transcription factors have been identified as key regulators of anthocyanin biosynthesis in various plant species [147]. MYC like bHLH proteins can physically interact with MYB transcription factor and WD40-repeat protein genes to form complexes, known as MBW complex, that control the transcript levels of genes by binding to their promoter regions, thereby regulating anthocyanin in biosynthesis [22,114], and these complexes are also crucial for the regulation of flavonoid pathway [115,116,117].

## 6. Conclusions and Future Prospects

As the second largest superfamily in Arabidopsis, most bHLH transcription factors are characterized by a signature domain, which consists of approximately 60 amino acids, with an N-terminal basic DNA binding domain and a C-terminal protein interaction domain. Although more than 160 genes have already been predicted to belong to the bHLHs family and are classified into 12 subfamilies [14], relatively few have been characterized according to current studies [13,148]. Recent research studies have demonstrated that bHLHs transcription factors are characterized by their roles in a broad range of plant growth and various developmental processes.

**Figure 2:**
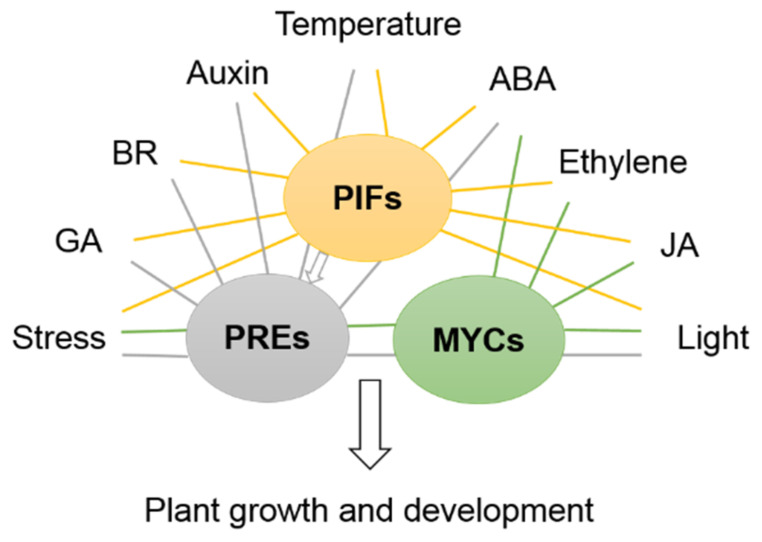
Simplified model for bHLH PREs, MYCs and PIFs, which play central roles in many pathways in Arabidopsis. PIFs bind to their target genes to regulate plant growth and development via manipulation of signaling pathways. PREs and MYCs also participate in core transcription networks together with PIFs to control plant responses to the environment.

Some transcription factors play the more crucial roles in many aspects of plant development, such as MYCs, PREs and PIFs, and these bHLHs are key transcriptional regulators in the phytohormone crosstalk pathway and multiple biosynthesis pathways. As JAZ targets, MYCs are involved in JA-mediated gene expression and the ABA signaling pathway; they also mediate ET biosynthesis and regulate stamen development and flowering time. PREs respond to GA, BR, temperature and light signaling to positively regulate cell elongation. PIFs are also involved in many other signaling pathways, such as in mediating pathways responsive to light, GA, IAA, ABA, ET and abiotic/biotic stress [73,149,150] (Figure 2).

Overall, in this review, we have compiled the current research on bHLH function and provided a relatively complete overview of the bHLH transcription factor family. This review enriches our understanding of this family and provides new insight into the mechanism by which bHLHs regulate various biological processes. However, there remain many aspects that have not been described because of the complicated crosstalk with other transcription factors. Our understanding of function the bHLH proteins have improved tremendously during the past several years, and the research in this area is well characterized. Future studies will further elucidate the mechanism of how bHLH proteins coordinate the multiple internal and external environments to regulate plant growth.

## Figures and Tables

**Table 1:** Functional characterized bHLH protein family.

Pathway	AGI Gene Code	Generic Name	Synonym	Functions Characterized	Group	Reference
Seed germination	At4g01460	bHLH57		Involved in seed dormancy process	Ia	[22,23]
	At2g20180	bHLH15	PIF1/PIL5	Negative regulator of phytochrome-mediated seed germination	VIIa	[24,25,26]
	At4g36930	bHLH24	SPT	Reduced seed dormancy	VIIb	[27,28]
Flowering	At1g35460	bHLH80	FBH1	Regulate the effect of CO flowering time	IX	[29]
	At4g34530	bHLH63	CIB1	Promote CRY2-dependent floral initiation	XII	[30,31,32]
	At5g65640	bHLH93	NFL	Involved in GA mediated control of flowering time	IIIb	[33]
	At1g32640	bHLH6	MYC2	Required in the JA pathway for regulating flowering time	IIIe	[34]
	At5g46760	bHLH5	MYC3	Required in the JA pathway for regulating flowering time	IIIe	[34]
	At4g17880	bHLH4	MYC4	Required in the JA pathway for regulating flowering time	IIIe	[34]
	At1g18400	bHLH44	BEE1	Regulate photoperiodic flowering	XII	[35]
	At2g43010	bHLH9	PIF4	Accelerate the flowering by activating FT at high temperature	XII	[36]
	At4g36930	bHLH24	SPT	Play a role in floral morphogenesis processes	VIIa	[37]
Cell fate	At1g66470	bHLH83	RHD6	ROOT HAIR DEFECTIVE6	VIIb	[13,38,39]
	At5g37800	bHLH86	RSL1	Partially redundant and involved in root hair development	VIIIc	[40]
	At4g33880	bHLH85	RSL2	Partially redundant and involved in root hair development	VIIIc	[40]
	At2g14760	bHLH84	RSL3	RHD6-LIKE 3, required for root-hair growth	VIIIc	[40,41,42]
	At1g27740	bHLH54	RSL4	Promote postmitotic cell growth in root-hair cells	VIIIc	[40,41,42]
	At5g58010	bHLH82	LRL3	Regulate root hair development.	XI	[40,41,42]
	At5g41315	bHLH1	GL3	Trigger the trichome initiation pathways	IIIf	[41,43,44]
	At1g63650	bHLH2	EGL3	Regulate trichome and root hair development	IIIf	[45,46]
	At5g46830	bHLH28	MYC5	Calcium-binding transcription factor involved in salt stress signaling	IIIe	[47]
	At1g32640	bHLH6	MYC2	Positive regulator of lateral root formation.	IIIe	[47]
	At5g46760	bHLH5	MYC3	Form the bHLH-MYB complex to regulate the stamen development	IIIe	[47]
	At4g17880	bHLH4	MYC4	form the bHLH-MYB complex to regulate the stamen development	IIIe	[47]
	At3g24140	bHLH97	FAMA	Promote differentiation of stomatal guard cells	Ia	[48]
Plant mineral nutrition and abiotic stress	At3g56970	bHLH38	ORG2	Regulate the Fe-deficiency response	Ib	[49]
	At3g56980	bHLH39	ORG3	Regulate the Fe-deficiency response	Ib	[49]
	At2g41240	bHLH100		A key regulator of iron-deficiency responses	Ib	[50]
	At5g04150	bHLH101		A key regulator of iron-deficiency responses	Ib	[50]
	At2g28160	bHLH29	FIT	Regulate iron uptake responses	IIIa	[49,50,51,52,53]
	At3g19860	bHLH121	URI	Act as an essential part of the iron deficiency signaling pathway	IVb	[54]
	At3g23210	bHLH34	IDT1	Involved in Fe regulation.	IVc	[55,56,57,58]
	At4g14410	bHLH104		Positively regulate Fe deficiency response	IVc	[55,56,57,58]
	At5g54680	bHLH105	ILR3	Plays an important role in Fe homeostasis	IVc	[55,56,57,58]
	At1g51070	bHLH115		Involved in response to Fe	IVc	[55,56,57,58]
	At3g47640	bHLH47	PYE	Regulate response to iron deficiency in Arabidopsis roots	IVb	[59,60,61,62]
	At4g36060	bHLH11		Basic helix-loop-helix (bHLH) DNA-binding superfamily protein	IVb	[63]
Stress response	At3g26744	bHLH116	ICE1	Improve cold tolerance through an ABA independent pathway	IIIb	[64]
	At3g06590	bHLH148	AIF2/RITF1	Involved in the detoxification of ROS which generated by salt stress	Orphans	[65]
	At1g61660	bHLH122		Mediate multiple response to improve stress tolerance	IX	[66,67]
	At2g43010	bHLH9	PIF4	Accelerate the flowering by activating FT at high temperature	XII	[68,69,70]
Light signaling	At2g46970	bHLH124	PIL1	Associated with APRR1/TOC1 and is a member of PIF3 family	VIIa	[24,25]
	At3g59060	bHLH65	PIF5/PIL6	Involved in shade avoidance	VIIa	[24,25]
	At3g62090	bHLH132	PIF6/PIL2	Associated with APRR1/TOC1 and is a member of PIF3 family	VIIa	[24,25]
	At1g09530	bHLH8	PIF3	Interact with photoreceptors phyA and phyB.	VIIa	[24,71,72]
	At2g43010	bHLH9	PIF4	Interact with active PhyB protein	VIIa	[24,25,73,74]
	At1g02340	bHLH26	HFR1	Involved in phytochrome signaling	VIIb	[75]
	At2g42870	bHLH165	PAR1	Control plant development and as a negative regulator of SAS	Orphans	[76,77,78]
	At3g58850	bHLH166	PAR2	Control plant development and as a negative regulator of SAS	Orphans	[76,77,78]
	At2g20180	bHLH15	PIF1/PIL5	A key negative regulator of phytochrome-mediated response	VIIa	[79,80,81,82]
	At5g61270	bHLH72	PIF7	Interacts specifically with Pfr form of phyB	VIIb	[83,84,85]
JA signaling	At1g32640	bHLH6	MYC2	Regulates diverse JA-dependent functions	IIIe	[86,87]
	At5g46760	bHLH5	MYC3	Act together with MYC2 and MYC4 to activate JA-responses	IIIe	[86,87]
	At4g17880	bHLH4	MYC4	Act together with MYC2 and MYC3 to activate JA-responses	IIIe	[86,87]
	At5g41315	bHLH1	GL3	Repressed by JAZs	IIIf	[88,89,90,91]
	At2g22750	bHLH18		Induced by JA and inhibit the transcription of the FIT	IVa	[92]
	At2g22760	bHLH19		Induced by JA and inhibit the transcription of the FIT	IVa	[92]
	At2g22770	bHLH20	NAI1	Induced by JA and inhibit the transcription of the FIT	IVa	[92]
	At4g37850	bHLH25		Induced by JA and inhibit the transcription of the FIT	IVa	[92]
IAA signaling	At1g59640	bHLH31	BPEp	Involved in the control of petal size	XII	[93,94]
	At1g26945	bHLH163	PRE6	Involved in ABA and salts responses	XV	[93,95]
ABA signaling	At1g61660	bHLH112		Mediate multiple response to improve stress tolerance	X	[96,97]
	At2g46510	bHLH17	AIB	Involved in response to ABA, repress MYC2-activated leaf senescence	IIId	[98,99]
	At1g32640	bHLH6	MYC2	Induced by dehydration stress, ABA and blue light	IIIe	[17]
	At2g43140	bHLH129		Regulate root elongation and ABA response.	IX	[100]
			PREs	Involved in the regulation of ABA and salt responses	XV	[101]
			BEEs	Repressed by ABA and responses to abiotic stress	Va	[102]
	At5g43650	bHLH92		Involved in salt and osmotic stress tolerance	IVd	[103]
Cross-talk between light and phyto-hormones	At5g39860	bHLH136	PRE1/BNQ1	Mediate brassinosteroid regulation of cell elongation	XV	[25,104,105]
	At5g15160	bHLH134	PRE2/BNQ3	PHD finger family protein	XV	[95]
	At3g47710	bHLH161	PRE4/BNQ3	Required for appropriate regulation of flowering time and regulating light responses.	XV	[106]
	At3g28857	bHLH164	PRE5	Involved in the regulation of the light, GA, BR signaling pathways	XV	[25,104]
	At1g26945	bHLH163	PRE6/KIDARI	Interacts with HFR1 and negatively regulates its activity.	XV	[95]
	At2g43060	bHLH158	IBH1	ILI1 binding bHLH 1	Orphans	[107,108]
	At2g18300	bHLH64	HBI1	Involved in positive regulation of cell elongation and proliferation	XII	[104]
	At2g43010	bHLH9	PIF4	Negatively regulate phyB mediated responses and involved in SAS	VIIa	[25,104]
	At1g18400	bHLH44	BEE1	Function in the early response to BRs	XII	[18,109]
	At4g36540	bHLH58	BEE2	Function redundant with BEE1/3	XII	[18,110]
	At1g73830	bHLH50	BEE3	Function redundant with BEE1/2	XII	[18]
	At5g08130	bHLH46	BIM1	BES1-INTERACTING MYC-LIKE 1, involved in BRs signaling	Va	[111,112]
	At1g69010	bHLH102	BIM2	Involved in brassinosteroid signaling and modulated SAS	Va	[111,112]
	At5g38860	bHLH141	BIM3	Involved in brassinosteroid signaling and modulated SAS	Va	[111,112]
Other aspects	At4g16430	bHLH3	JAM3	Repress MYC2-activated leaf senescence, negatively regulate JA response	IIId	[47]
	At1g01260	bHLH13	JAM2/MYC7E	Repress MYC2-activated leaf senescence, negatively regulate JA response	IIId	[47]
	At4g00870	bHLH14		Repress MYC2-activated leaf senescence, negatively regulate JA responses	IIId	[47]
	At2g46510	bHLH17	AIB	Involved in response to ABA, repress MYC2-activated leaf senescence	IIId	[47]
			PIFs	Promoted leaf senescence	VIIa	[113]
			MYCs	Positive regulator of Positively regulates flavonoid biosynthesis	IIIe	[67,114,115,116,117]

## Data Availability

Data sharing not applicable. No new data were created or analyzed in this study. Data sharing is not applicable to this article.
